# Improving health literacy in a Japanese community population—A pilot study to develop an educational programme

**DOI:** 10.1111/hex.12678

**Published:** 2018-03-30

**Authors:** Hirono Ishikawa, Ikuko Yamaguchi, Don Nutbeam, Mio Kato, Tsuyoshi Okuhara, Masafumi Okada, Takahiro Kiuchi

**Affiliations:** ^1^ Department of Health Communication School of Public Health The University of Tokyo Tokyo Japan; ^2^ Approved Specified Nonprofit Organization COML Osaka Japan; ^3^ School of Public Health University of Sydney Sydney NSW Australia

**Keywords:** health communication, health education, health literacy

## Abstract

**Objective:**

Although a growing number of interventional studies on health literacy have been conducted recently, the majority were designed in clinical settings, focusing mainly on functional health literacy. This study evaluated a programme designed to improve health literacy in a community population, with a scope of going beyond functional health literacy.

**Methods:**

In collaboration with an Approved Specified Nonprofit organization (NPO), we evaluated a five‐session programme designed to provide basic knowledge on health‐care policy and systems, current issues in health care in Japan, patient roles and relationships with health‐care providers and interpersonal skills. In total, 67 of 81 programme participants agreed to participate in the study, and 54 returned the completed questionnaires at baseline and at follow‐up. Health literacy and trust in the medical profession were measured at baseline and at follow‐up. Participants’ learning through the programme was qualitatively analysed by thematic analysis.

**Results:**

Quantitative examinations of the changes in health literacy and degree of trust in medical professionals between the baseline and follow‐up suggested that health literacy significantly improved after implementing the programme. The thematic analysis of participants’ learning throughout the programme suggested that they not only acquired knowledge and skills but also experienced a shift in their beliefs and behaviours.

**Discussion:**

Providing individuals who are motivated to learn about health‐care systems and collaborate with health‐care providers with the necessary knowledge and skills may improve their health literacy, which could enable them to maintain and promote their health and that of their family and other people around them.

## 1 INTRODUCTION

Over the past few decades, health literacy has gained an increasing amount of attention as a factor related to various health behaviours and outcomes. Health literacy represents “the cognitive and social skills that determine the motivation and ability of individuals to gain access to, understand, and use information in ways that promote and maintain good health.”^1^ It implies the achievement of a level of knowledge and confidence, as well as personal skills that allow action to be taken to improve personal and community health by changing personal lifestyles and living conditions. These skills can be measured and vary from individual to individual.

These differences in skills have been categorized as *functional*,* interactive* and *critical health literacy*.^2^ Such a classification was derived from mainstream literacy studies and has the advantage of signalling the impact that differences in skill levels may have on health‐related decisions and actions. *Functional health literacy* describes basic skills that are sufficient for individuals to obtain relevant health information (e.g. on health risks and on how to use the health system) and to be able to apply that knowledge to a range of prescribed activities. *Interactive health literacy* describes more advanced literacy skills that enable individuals to extract health information and derive meaning from different forms of communication, apply new information to changing circumstances and engage in interactions with others to extend the information available and make decisions. *Critical health literacy* describes the most advanced literacy skills that can be applied to critically analyse information from a wide range of sources and information related to a greater range of health determinants, as well as to use this information to exert greater control over life events and situations that impact their health.

Health literacy can be improved by providing information, effective communication and a structured education.^3^ With growing evidence of the relationship between inadequate health literacy and poor health outcomes, interventional research has been conducted with the aim of resolving problems related to inadequate health literacy. The majority of such interventions have been conducted in clinical settings, focusing mainly on functional health literacy.^4^, ^5^, ^6^, ^7^, ^8^, ^9^ These studies have provided evidence that individuals with lower health literacy can be identified and supported to develop a better understanding of and skills to improve health behaviours and outcomes. On the other hand, fewer interventions have been conducted in community settings, and less emphasis has been placed on improving higher level interactive and critical health literacy vs functional health literacy.^3^, ^4^

The Japanese have one of the world's longest life expectancies; however, a previous study suggested that health literacy in the Japanese general population is lower than that in European countries.^10^ They suggested that part of the reason was the lack of a comprehensive website for reliable health information comparable to MedlinePlus (US National Library of Medicine) and the inefficiency of the Japanese primary health‐care system, which lacks general practitioners as gatekeepers. Universal health coverage in Japan allows all citizens free access to health‐care services.^11^ However, there have been few opportunities for the general public to acquire basic information concerning the health‐care system, available health‐care resources and skills to effectively interact with health‐care providers. These skills and abilities correspond to the higher level interactive and critical health literacy skills described above,^2^ which better enable individuals to utilize health‐care information and services, collaborate with health‐care providers, and engage in healthy choices and behaviours within the context of the health‐care systems in their society.

Individuals with lower health literacy or education level are more likely to have unrealistic expectations regarding health care, often exacerbated by difficulties in obtaining and understanding health information.^12^, ^13^ This can often lead to dissatisfaction with the health‐care system and in some cases growing distrust of health‐care professionals.^14^, ^15^ Effective patient education and improved health literacy could lead to the modification of such unrealistic expectations and may also help foster improved trust in health‐care professionals.^16^, ^17^, ^18^

Furthermore, health literacy is not solely an individual skill but is also a distributed resource available within an individual's social entourage.^19^ A previous study suggested that health literacy is distributed through family and social networks, and individuals often draw on the health literacy skills of others to seek, understand and use health information.^20^ This may be especially true in Asian culture in a collective context.^21^

In this study, we evaluated an educational programme to develop higher level health literacy (i.e. interactive and critical health literacy) through the acquisition of improved knowledge about current health‐care systems, and interpersonal skills that enhance patient‐provider relationships. We used a mixed method approach to examine changes in health literacy and trust in the medical profession before and after the programme, and explored what the participants had learned through the programme using thematic analysis.

## 2 METHODS

### 2.1 Interventional programme

The programme was originally developed in 2009 by an Approved Specified Nonprofit organization (NPO) in Japan that advocates for patient empowerment and collaboration with health‐care providers. In collaboration with this NPO, we provided a five‐session programme. Each session was 3 hours in duration and consisted of lectures, discussions and role‐playing exercises.

The contents of the programme are shown in Table 1. The contents were developed based on the needs among patients and citizens identified through the NPO's experiences with a telephone counselling service and seminars on health care for the general public for more than 20 years. The authors, who are public health researchers, reviewed the programme and linked the contents with the concept of health literacy. The programme was intended to provide basic knowledge about health‐care systems in Japan and how to find health‐care services when needed (considered as a part of *functional and interactive health literacy*), to develop active patient roles in the relationships with health‐care providers and effective communication skills (considered as a part of *interactive health literacy*), to consider current issues in health care in Japan and to facilitate successful patient/citizen collaboration with health‐care providers to improve the health care (considered as a part of *critical health literacy*).

**Table 1:** Content of the intervention programme

Session	Programme contents
1) Introduction	Overview of the programme Self‐introduction of participants Introduction of the NPO activities Needs for patient and citizen participation in health care: possible volunteer opportunities
2) Basics of the Japanese health‐care system	History of health care in Japan (systems, some historical events, development of patient's right) Types of health‐care institutions and professionals (features and roles) Basics of health‐care service and insurance system Current issues in health‐care practice (patient safety, clinical training systems, shortage and poor distribution of physicians, emergency care crisis, co‐operation between clinics and hospitals, etc.)
3) Patient experiences in health care	Troubles and difficulties experienced by patients in the health care: the data from the telephone counselling service Information and support sought by patients and family Shifts in patients’ attitude and current issues in patient‐provider relationship Communication and interpersonal skills in health care
4) Tips for smart patients	Tips for smart patients (how to choose medical institutions, get ready for your medical visit) What is second opinion? Basic knowledge about medial service fees (medical service fees system, health insurance coverage, out‐of‐pocket expenses, etc.)
5) Health care–related laws and systems	Health care–related laws and systems (ways to address complaints, personal information protection laws, the adult guardianship system, medical expenses deductions) Basic information about medications (clinical trials, generics, separation of medical and dispensary services, adverse effects)

All sessions were led by the chief director of the NPO who had been working with this NPO for about 25 years. A textbook for each class was distributed, and PowerPoint presentations were used for the lectures. These materials were revised every year only if there were any changes in the health‐care systems and statistical data, so that there were no significant changes in the programme contents among the waves.

### 2.2 Study design and participants

This study included the programmes provided in 2012 (two waves), 2015 (two waves) and 2016 (three waves). The programmes were provided either at the NPO's office building or at a seminar room of the university. The programme participants were recruited via the NPO newsletter, their website and social media, as well as using a leaflet. In total, 81 participants applied for the programme. Enrolment varied between 6 and 18 participants per wave. At the beginning of the programme, prospective participants were invited to take part in the study, and baseline questionnaires and consent forms were distributed. They were informed both orally and in writing that participation was voluntary and a 500‐yen (US$5) gift certificate would be sent in return for participation. In total, 67 participants returned completed consent forms and questionnaires (response rate: 82.7%). After completion of the programme, a follow‐up questionnaire was sent to each participant to be returned within a month; 54 respondents returned the completed questionnaire (follow‐up rate: 80.6%). We excluded two participants, who missed more than two sessions, from the analyses. Among the remaining participants, the rate of attendance of all five sessions was 82.7% (n = 43).

### 2.3 Measures

#### 2.3.1 Health literacy

Health literacy was measured using the Communicative and Critical Health Literacy Scale,^22^ at baseline and at follow‐up. This scale is based on the dimensions of health literacy described earlier^1^ and consists of five items addressing whether participants are able to: (i) collect health information from various sources, (ii) extract the information they want, (iii) understand and communicate the information obtained, (iv) consider the credibility of the information, and (v) make decisions based on the information in the context of health issues. Each item is rated on a 5‐point scale ranging from 1 (*strongly disagree*) to 5 (*strongly agree*). Scores for the items of each scale were summed and divided by the number of items in that scale to yield a scale score (theoretical range: 1‐5). The Cronbach's α value of the scale was 0.87.

#### 2.3.2 Trust in the medical profession

Trust in the medical profession was measured using an abbreviated, five‐item measure of patient trust in the medical profession validated by Dugan et al^23^. Each item was scored on a 5‐point Likert scale ranging from 1 (*strongly disagree*) to 5 (*strongly agree*) and summed to yield a scale score. The Cronbach's α value of the scale was 0.74.

#### 2.3.3 Learning throughout the programme

In the follow‐up questionnaire, the participants were asked what they had learned or gained through the programme using a free‐answer question. A thematic analysis approach was used to analyse these text‐based answers according to the steps proposed by Braun and Clarke^24^. The first author read and re‐read all answers to gain familiarity with the data, and then generated the initial codes. Then, the codes were collated into potential themes and subthemes. Through discussions with the co‐authors, the themes were reviewed, and a thematic map of the analysis was generated. Finally, an ongoing analysis was conducted to refine the specifics of each theme and identify the overall “story” of the analysis. The final results of the analysis were reviewed by the co‐authors to ensure that the findings were credible. To illustrate each theme, quotes were selected, based on their representativeness and/or illustrative power, and presented in the results section.

#### 2.3.4 Other variables

In the baseline questionnaire, we also collected data on sociodemographic characteristics, including age, gender, and educational attainment, routine medical visit (once in 3 months or more), possession of a health care–related license or work experience, and volunteer experience in health care.

### 2.4 Statistical analyses

Scales measuring changes in health literacy and the degree of trust in the medical profession, between baseline and follow‐up, were examined using the paired *t* test. Analyses stratified by participant characteristics, such as gender and health care–related license or work experience status, were also conducted. Analyses were carried out using Stata software (ver. 14.2; StataCorp LP, College Station, TX, USA).

## 3 RESULTS

### 3.1 Participant characteristics

Table 2 shows the characteristics of the study participants. They ranged in age from 24 to 78 years (mean, 54.9 years; standard deviation [SD], 11.1 years) and more than 70% were female. A total of 62.6% of the participants were university graduates or above, which was much higher than the proportion among the general population in Japan. Sixteen participants (23.9%) had a health care–related license or experience with health‐care work, including as home helpers, social workers, nurses, pharmacists and dietitians. In addition, 18 participants had experience with volunteering in a health‐care setting, including working in a patient library, providing patient support at outpatient services, coordinating a peer support group and acting as a simulated patient.

**Table 2:** Participant characteristics (N = 65)

	N	%
Gender		
Male	18	26.9
Female	47	70.1
Age		
Mean, SD	54.7	11.2
Highest level of education		
Junior high school	1	1.5
High school	5	7.5
2‐year college	19	28.4
University	33	49.3
Graduate school	7	10.4
Routine medical visit	40	59.7
Health care–related license/work	15	22.4
Volunteer experience in health care	18	26.9

### 3.2 Quantitative results

As shown in Table 3, the health literacy score improved significantly after the programme, whereas there was no significant change in the degree of trust in the medical profession. Stratified analyses suggested that there was no significant interaction between participant characteristics, such as gender, and health care–related experiences.

**Table 3:** Changes in health literacy and trust in medical profession scores (N = 52)

	Mean	SD	*P*‐value^a^
Health literacy			
Baseline	3.67	0.75	<.001
Follow‐up	3.93	0.62	
Trust in medical profession			
Baseline	3.13	0.67	.616
Follow‐up	3.09	0.66	

a Paired *t* test.

### 3.3 Qualitative results

**Figure 1:**
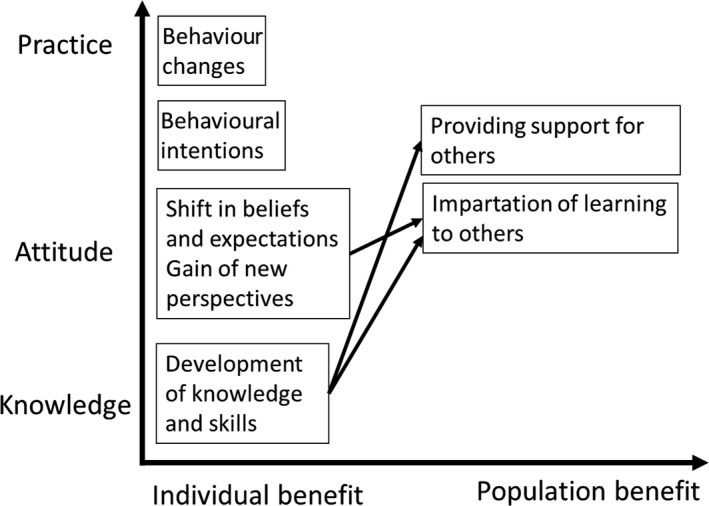
Thematic analyses of learning: level of learning and perspectives

All 52 participants who returned the follow‐up questionnaire provided an answer to the free‐answer question regarding what they had learned or gained through the programme. Figure 1 shows a summary map of the themes of learning at different levels, which included the following: (i) knowledge and skills, (ii) attitudes, (iii) behaviours and (iv) broadening of perspective from individual to population benefits.

#### 3.3.1 Knowledge and skills

The majority of participants stated that they had acquired some of the basic knowledge of the Japanese health‐care system, interpersonal and communication skills, the gathering of health information, and volunteer work in health care. These learning outcomes constituted the main areas that the programme was intended to cover.

[Health‐care systems]

I have a good understanding of the current healthcare system in Japan (female, 47).

I understand how to read statements regarding medical expenses and prescriptions (female, 63).

[Interpersonal and communication skills]

Attentive listening is always important for building relationships (male, 47).

I learned the importance of looking at oneself from a third person's viewpoint (female, 71).

When providing consultation, we should first try to understand the situation of the client and then collaborate regarding the process to find solutions (male, 63).

[Gathering health information]

Reading the newspaper: I realized that newspapers provide easy‐to‐understand and useful information of health‐related issues (female, 59).

Where to find various health information (female, 63).

[Volunteer work in health care]

Volunteers, such as simulated patients, are needed in medical education. (female, 50)

There is a greater variety of volunteer opportunities in healthcare than I had expected (female, 49).

#### 3.3.2 Attitude

Changes in participants’ beliefs and expectations regarding patient‐provider relationships and the role of patients were also commented by more than half of those who responded. The shift was seen in two ways: a more realistic understanding and positive perception of health‐care providers, and a less dependent attitude in their relationship with health‐care providers acknowledging a more active patient role.

[Patient‐provider relationship]

For me, physicians were a “black box” in the medical world. But now, I have found that they are “human beings,” just like us (female, 54).

A question raised after this program was whether patients and physicians could have common ground. The best medical treatment from a patient's perspective might not be the same as that from a healthcare provider's perspective. If there are such differences, good communication and building trust would be very difficult (male, 56).

Although I have been working as a caregiver and always told my clients the importance of collaboration, I had never thought that physicians and patients could collaborate in the healthcare process. This was a big surprise (female, 63).

[Role of the patient]

Before this program, I never had a distrust of physicians, and believed that I could rely on them and everything would go well. But, I have learned that it is myself, not the physician, who is responsible for my body and health (female, 63).

I learned that it is important for patients to have the ability to effectively communicate their specific situations, and try to learn what they do not understand. (female, 59)

In addition, some of the participants suggested that they were motivated to learn more, as they found there were many things of which they had previously been unaware.

[Motivation to learn]

I found that there are many things I do not know. I now understand the importance of trying to understand, even a medical expenses’ receipt (female, 58).

It is important to have an interest in health and to try and acquire knowledge before we become patients (female, 57).

#### 3.3.3 Behaviours

Actual changes in behaviours or intentions to change behaviours were mentioned by a few participants.

[Behavioural intentions]

I would like to make use of what I have learned when I receive medical care in the future. I would like to be considerate of healthcare staff and build a good relationship with them (female, 50).

[Behaviours]

After I heard this lecture, I felt like writing a letter to thank my doctor who had been taking care of me. I wrote him a letter and I feel that our relationship will change for the better (female, 48).

I became conscious of the time when making a medical visit (male, 78).

#### 3.3.4 Broadening of perspective from individual to population benefits

Some participants stated their intention to impart what they had learned to, and provide support for, others. These comments might suggest that the benefits related to the participants’ learning might spread from the participants themselves to other people around them.

[Impartation of learning to others]

I want more people to undertake this program. It should be introduced into compulsory education (female, 34).

We are living in an age of increasing medical care needs. I would like to pass on the resource “10 tips when visiting a doctor” to my friends and acquaintances (female, 50),

[Providing support for others]

The information I have learned will also be useful if members of my family become sick and must visit a physician (male, 48).

I would like to participate in some volunteer work in healthcare (female, 57).

## 4 DISCUSSION

This pilot study quantitatively and qualitatively evaluated a programme to improve health literacy in a community population, focusing on aspects of health literacy beyond functional health literacy. As expected, our quantitative analyses suggested that participants’ health literacy scores significantly improved after the programme. Few interventional studies have attempted to improve higher level health literacy (i.e. interactive and critical health literacy).^3^ The findings of our qualitative analysis suggest that participants obtained basic knowledge and skills concerning health‐care systems, interpersonal and communication skills, and the seeking of information, which were precisely the learning points that this programme was designed to offer. In addition, the participants acquired not only knowledge and skills, but also experienced a shift in beliefs and behaviours, with respect to collaborating with health‐care providers and effectively utilizing health‐care services as active participants.

Importantly, some participants also mentioned supporting, and imparting their knowledge to, others. Previous studies have suggested that individuals with higher health literacy may influence and support others towards engaging in healthy choices and behaviours.^20^, ^25^, ^26^ Health literacy is a product of both an individual's capabilities and the demands of the population to which they belong including the health‐care systems, culture and social capital.^27^, ^28^, ^29^ Improving health literacy among a population not only reduces the demand for health literacy for individual members of that population, but also moderates the relationship between individual health literacy and health outcomes by providing support for individuals seeking to understand information, make health decisions and engage in self‐management.^30^ Providing individuals who are motivated to learn about health‐care systems and collaborate with health‐care providers with the necessary knowledge and skills may enable them to maintain and promote their health, and that of their family and other people around them.

In contrast with the generally positive outcomes regarding health literacy, trust in the medical profession did not significantly change after completing the programme. It is argued that adequate health literacy is essential for effective patient interactions with physicians and that this would be associated with higher levels of trust in physicians.^31^ However, previous studies have reported both positive and negative, as well as a complete lack of significant associations between health literacy and degree of trust in physicians.^32^, ^33^, ^34^ This may be partly because the relationship between health literacy and trust is not linear. Less health literate patients may be more inclined to trust without questioning the recommendations of their physician.^13^, ^33^ Thus, extremely high trust may sometimes indicate a dependence on physicians because of a lack of self‐confidence. Although there are no data concerning what level of trust is “adequate,” our qualitative results suggest that participants gained a more realistic and positive understanding of health‐care providers, which might lead to higher trust, while they also learned to take a less dependent attitude in their relationship with physicians. Alternatively, trust may change after actual experiences of interacting with health‐care professionals. A previous study suggested that highly health literate patients who had completed a patient education programme faced difficulties interacting with their health‐care providers because of the power imbalance and lack of respect for their expertise.^17^ Further investigation is needed to explore the relationship between health literacy and trust in the medical profession.

### 4.1 Limitations and suggestions for further research

Our study was not without limitations. First, this study was a pilot intervention with a relatively small sample size. Although we found significant improvements in health literacy post‐intervention, the lack of a comparative group limits the interpretation of our findings. Also, participant characteristics (such as gender, age and health care–related work experience) might have had moderating effects on the changes. However, due to the small sample size, we might not have been able to fully examine such moderating effects by the participant characteristics. Further studies with more sophisticated designs with larger sample size are warranted to confirm our findings. Second, the participants were all volunteers and relatively well‐educated and motivated to learn about health care. The mean score of baseline health literacy was 3.67, which was somewhat higher than that of a previous nationwide online survey of the Japanese general population (N = 712; mean ± SD, 3.59 ± 0.62),^35^ but lower than a study of Japanese male office workers who were all university graduates (N = 190; mean ± SD, 3.72 ± 0.68).^22^ The generalizability of our findings to the general population is debatable. Future studies should carefully consider whether this programme can also be applied to those with less motivation and/or lower health literacy or should target those with relatively higher motivation and health literacy who could act as a support for those around them. Third, health literacy was measured based on a self‐reported questionnaire. The responses should be considered the participants’ perceptions about their capabilities, which might be different from actual knowledge and skills. Fourth, the programmes were provided in multiple waves from 2012 to 2016. It is possible that there have been societal changes in the perception of the health‐care profession during these years. In additions, although we tried to keep the educational contents identical across the waves, there might have been unintentional differences among the waves. At least, however, when stratified by the year, the improvement in health literacy was consistently observed in each stratum, if not statistical significant due to the small sample size. Last, future evaluations of the intervention should also include a longer follow‐up period to determine to what extent improvements in health literacy are retained.

## 5 CONCLUSIONS

Despite these limitations, this is one of the first studies to evaluate a programme designed to improve health literacy in a community population, focusing on higher level health literacy, that is beyond functional health literacy and trust in the medical profession. Our findings suggest that this type of interventional programme might have potential to improve health literacy in the general population. During the programme, participants perceived that they had acquired not only knowledge and skills, but also experienced a shift in their beliefs and behaviours. Providing individuals who are motivated to learn about health‐care systems and collaborate with health‐care providers with the necessary knowledge and skills may enable them to maintain and promote their health, and that of their family and other people around them.

## CONFLICT OF INTEREST

The authors declare no conflict of interest.

## References

[1] Nutbeam D . Health promotion glossary. Health Promot Int. 1998;13:349‐364.

[2] Nutbeam D . Health literacy as a public health goal: a challenge for contemporary health education and communication strategies into the 21st century. Health Promot Int. 2000;15:259‐267.

[3] Nutbeam D , McGill B , Premkumar P . Improving health literacy in community populations: a review of progress. Health Promot Int. 2017; Epub ahead of print. 10.1093/heapro/dax015. 28369557

[4] Barry MM , D'Eath M , Sixsmith J . Interventions for improving population health literacy: insights from a rapid review of the evidence. J Health Commun. 2013;18:1507‐1522. 24298885 10.1080/10810730.2013.840699

[5] Dennis S , Williams A , Taggart J , et al. Which providers can bridge the health literacy gap in lifestyle risk factor modification education: a systematic review and narrative synthesis. BMC Fam Pract. 2012;13:44. 22639799 10.1186/1471-2296-13-44 PMC3515410

[6] Manafo E , Wong S . Health literacy programs for older adults: a systematic literature review. Health Educ Res. 2012;27:947‐960. 22752153 10.1093/her/cys067

[7] Schaefer CT . Integrated review of health literacy interventions. Orthop Nurs. 2008;27:302‐317. 18832992 10.1097/01.NOR.0000337283.55670.75

[8] Sheridan SL , Crespo E . Does the routine use of global coronary heart disease risk scores translate into clinical benefits or harms? A systematic review of the literature. BMC Health Serv Res. 2008;8:60. 18366711 10.1186/1472-6963-8-60 PMC2294118

[9] Taggart J , Williams A , Dennis S , et al. A systematic review of interventions in primary care to improve health literacy for chronic disease behavioral risk factors. BMC Fam Pract. 2012;13:49. 22656188 10.1186/1471-2296-13-49 PMC3444864

[10] Nakayama K , Osaka W , Togari T , et al. Comprehensive health literacy in Japan is lower than in Europe: a validated Japanese‐language assessment of health literacy. BMC Public Health. 2015;15:505. 26001385 10.1186/s12889-015-1835-x PMC4491868

[11] Ikegami N , Yoo BK , Hashimoto H , et al. Japanese universal health coverage: evolution, achievements, and challenges. Lancet. 2011;378:1106‐1115. 21885107 10.1016/S0140-6736(11)60828-3

[12] Magasi S , Durkin E , Wolf MS , Deutsch A . Rehabilitation consumers’ use and understanding of quality information: a health literacy perspective. Arch Phys Med Rehabil. 2009;90:206‐212. 19236975 10.1016/j.apmr.2008.07.023

[13] Smith SK , Dixon A , Trevena L , Nutbeam D , McCaffery KJ . Exploring patient involvement in healthcare decision making across different education and functional health literacy groups. Soc Sci Med. 2009;69:1805‐1812. 19846245 10.1016/j.socscimed.2009.09.056

[14] Bell RA , Kravitz RL , Thom D , Krupat E , Azari R . Unmet expectations for care and the patient‐physician relationship. J Gen Intern Med. 2002;17:817‐824. 12406352 10.1046/j.1525-1497.2002.10319.x PMC1495125

[15] Coran JJ , Koropeckyj‐Cox T , Arnold CL . Are physicians and patients in agreement? Exploring dyadic concordance. Health Educ Behav. 2013;40:603‐611. 23345336 10.1177/1090198112473102

[16] Lateef F . Patient expectations and the paradigm shift of care in emergency medicine. J Emerg Trauma Shock. 2011;4:163‐167. 21769199 10.4103/0974-2700.82199 PMC3132352

[17] Snow R , Humphrey C , Sandall J . What happens when patients know more than their doctors? Experiences of health interactions after diabetes patient education: a qualitative patient‐led study. BMJ Open. 2013;3:e003583. 10.1136/bmjopen-2013-003583 PMC3831109 24231459

[18] Woolf SH . The price of false beliefs: unrealistic expectations as a contributor to the health care crisis. Ann Fam Med. 2012;10:491‐494. 23149524 10.1370/afm.1452 PMC3495921

[19] Papen U . Literacy, learning and health ‐ A social practices view of health literacy. Lit Numer Stud. 2009;16:19‐34.

[20] Edwards M , Wood F , Davies M , Edwards A . ‘Distributed health literacy’: longitudinal qualitative analysis of the roles of health literacy mediators and social networks of people living with a long‐term health condition. Health Expect. 2015;18:1180‐1193. 23773311 10.1111/hex.12093 PMC5060848

[21] Dutta MJ , Kaur S , Luk P , Lin J , Lee ST . Health information seeking among Singaporeans: roles and collective contexts. Health Commun. 2017;33:433‐442. 28151015 10.1080/10410236.2016.1278493

[22] Ishikawa H , Nomura K , Sato M , Yano E . Developing a measure of communicative and critical health literacy: a pilot study of Japanese office workers. Health Promot Int. 2008;23:269‐274. 18515303 10.1093/heapro/dan017

[23] Dugan E , Trachtenberg F , Hall MA . Development of abbreviated measures to assess patient trust in a physician, a health insurer, and the medical profession. BMC Health Serv Res. 2005;5:64. 16202125 10.1186/1472-6963-5-64 PMC1262715

[24] Braun V , Clarke V . Using thematic analysis in psychology. Qual Res Psychol. 2006;3:77‐101.

[25] Arozullah AM , Lee SY , Khan T , et al. The roles of low literacy and social support in predicting the preventability of hospital admission. J Gen Intern Med. 2006;21:140‐145. 16336616 10.1111/j.1525-1497.2005.00300.x PMC1484663

[26] Lee SY , Arozullah AM , Cho YI . Health literacy, social support, and health: a research agenda. Soc Sci Med. 2004;58:1309‐1321. 14759678 10.1016/S0277-9536(03)00329-0

[27] Baker DW . The meaning and the measure of health literacy. J Gen Intern Med. 2006;21:878‐883. 16881951 10.1111/j.1525-1497.2006.00540.x PMC1831571

[28] Nutbeam D . The evolving concept of health literacy. Soc Sci Med. 2008;67:2072‐2078. 18952344 10.1016/j.socscimed.2008.09.050

[29] Zarcadoolas C . Advancing Health Literacy: A Framework for Understanding and Action. San Francisco, CA: Jossey‐Bass; 2006.

[30] Ishikawa H , Yano E . Patient health literacy and participation in the health‐care process. Health Expect. 2008;11:113‐122. 18494956 10.1111/j.1369-7625.2008.00497.x PMC5060442

[31] Gupta C , Bell SP , Schildcrout JS , et al. Predictors of health care system and physician distrust in hospitalized cardiac patients. J Health Commun. 2014;19(Suppl 2):44‐60. 25315583 10.1080/10810730.2014.934936 PMC4318514

[32] Aboumatar HJ , Carson KA , Beach MC , Roter DL , Cooper LA . The impact of health literacy on desire for participation in healthcare, medical visit communication, and patient reported outcomes among patients with hypertension. J Gen Intern Med. 2013;28:1469‐1476. 23690237 10.1007/s11606-013-2466-5 PMC3797328

[33] White RO , Osborn CY , Gebretsadik T , Kripalani S , Rothman RL . Health literacy, physician trust, and diabetes‐related self‐care activities in Hispanics with limited resources. J Health Care Poor Underserved. 2013;24:1756‐1768. 24185168 10.1353/hpu.2013.0177 PMC3916094

[34] Rodriguez V , Andrade AD , Garcia‐Retamero R , et al. Health literacy, numeracy, and graphical literacy among veterans in primary care and their effect on shared decision making and trust in physicians. J Health Commun. 2013;18(Suppl 1):273‐289. 24093361 10.1080/10810730.2013.829137 PMC3815195

[35] Ishikawa H , Kato M , Kiuchi T . Associations of health literacy and information sources with health‐risk anxiety and protective behaviors. J Commun Healthc. 2016;9:33‐39.

